# Differential prevalence and associations of overweight and obesity by gender and population group among school learners in South Africa: a cross-sectional study

**DOI:** 10.1186/s40608-017-0165-1

**Published:** 2017-07-17

**Authors:** Sarah Negash, Charles Agyemang, Tandi E Matsha, Nasheeta Peer, Rajiv T Erasmus, Andre P Kengne

**Affiliations:** 1 Department of Public Health, Academic Medical Center, University of Amsterdam, Amsterdam, The Netherlands; 2 Department of Biomedical Technology, Faculty of Health and Wellness Sciences, Cape Peninsula University of Technology, Cape Town, South Africa; 3 Non-Communicable Diseases, Research Unit, South African Medical Research Council, Cape Town, South Africa; 4 Department of Medicine, University of Cape Town, Cape Town, South Africa; 5 Division of Chemical Pathology, Faculty of Health Sciences, University of Stellenbosch, Cape Town, South Africa

**Keywords:** Excess weight, Paediatrics, Cardio-metabolic comorbidities, Cardiovascular diseases

## Abstract

**Background:**

Factors influencing the increasing prevalence of overweight/obesity among children and adolescents in sub-Saharan Africa remain unclear. We assessed the prevalence and determinants of overweight and obesity and effects on cardio-metabolic profile in school learners in the Western Cape, South Africa.

**Methods:**

Cross-sectional data were collected from 7 to 18-year-old South African school learners attending 14 schools, randomly selected from 107 government schools in the areas. The learners were selected through stratified random sampling techniques. Logistic regressions were used to assess the determinants of overweight/obesity and its association with cardio-metabolic profile.

**Results:**

Among the 1559 participants, the overall prevalence of overweight/obesity was 22.9%. Being a girl (Odds ratio 2.51, 95% CI: 1.92–3.29), or Black African (1.35, 1.04–.75) was associated with increased odds of being overweight/obese. The identified health consequences among the overweight/obese learners differed between the ethnic groups. Overweight/obese coloured (mixed ancestry) learners were more likely to have hypertension (3.27, 1.18–9.08), hypertriglyceridemia (1.94, 0.99–3.78) and low high-density lipoprotein cholesterol (HDL-C) (3.65, 2.33–5.72), overweight/obese Black African learners had higher odds for hypertension (3.62, 1.31–10.04) and low HDL-C (1.56, 1.01–2.40) and overweight/obese White learners were prone to low HDL-C (5.04, 1.35–18.80).

**Conclusions:**

Overweight/obesity is highly prevalent among school learners in Western Cape (South Africa), with being female or Black African increasing the odds. That overweight/obesity is also associated with adverse cardio-metabolic risk profile aggravates the problem and suggests worse cardiovascular outcomes in South African young adults in the future.

**Electronic supplementary material:**

The online version of this article (doi:10.1186/s40608-017-0165-1) contains supplementary material, which is available to authorized users.

## Background

There has been a rapid rise in the prevalence of overweight/obesity with the disorder now affecting both developed and developing regions. Furthermore, the late twentieth century witnessed the emergence of childhood obesity, first in affluent nations and then in increasing numbers in poorer countries [1–3]. Childhood obesity has emerged as an equally challenging problem in developing as in developed regions [4]. In South Africa, the rate of overweight/obesity in children is at least as comparable to levels shown more than a decade ago in some developed countries, and may even be on par with many others [5].

The burden of overweight/obesity in children cannot be ignored because of its potentially devastating consequences, particularly its contribution to cardio-metabolic comorbidities and cardiovascular diseases (CVDs). Obesity in childhood and adolescence, as in adults, is associated with higher risk for the development of insulin resistance, type 2 diabetes mellitus, high systolic and diastolic blood pressures (BPs), dyslipidaemia [raised low-density lipoprotein cholesterol (LDL-C), increased triglycerides and low high-density lipoprotein cholesterol (HDL-C)], among other abnormalities detected [5, 6]. Consequently, childhood obesity is now regarded as one of the most serious public health challenges of the twenty-first century [7].

Similar to adults, the development of overweight/obesity in childhood is a complex and multifaceted interplay between genetic, epigenetic, environmental and behavioural factors with individual differences determining the degree of susceptibility to obesity [8]. Factors that influence susceptibility to an obesity-conducive environment include environmental dynamics, lifestyle choices, and cultural and sociologic factors, in addition to a genetic predisposition [9, 10]. These influences determine behaviours such as the consumption of energy-dense food that are high in fats and refined sugars, the levels of physical inactivity and the extent of sedentary behaviours that lead to the development of overweight/obesity in both children and adults [5]. Modifications in diet and physical activity, as well as addressing social and environmental factors, will curb the uptake of excess weight and may avert its serious health consequences.

The availability of local data on the prevalence, determinants and comorbidities of childhood obesity would have important implications for the provision of healthcare and for prevention strategies. This information, unique to the population being targeted, would assist in appropriate health service planning and be invaluable for the implementation of effective, population-specific, and gender- and age-orientated obesity prevention and management programmes.

Therefore, this study aimed to assess the prevalence, socio-demographic and behavioural determinants, and cardio-metabolic comorbidities of overweight and obesity among school learners in the Western Cape, South Africa.

## Methods

### Data collection and secondary data analysis

#### Study population

The study population consisted of 7–18-year-old learners attending government funded primary and secondary schools in the Western Cape between January 2007 and March 2008. Participants from the previously defined official South African population groups were included and comprised Black/African, Coloured and White learners. The Coloured group include people of Khoisan, Malaysian, Griqua, African and White descent and describe people of mixed ancestry [11]. Unlike the national demographics where Blacks comprise 80% of the population [12], in the Western Cape, Coloureds predominate at 49%, followed by Blacks (33%) and White (17%) [13]. Indians/Asians, the fourth South African population group comprise only 1% of the Western Cape population and were therefore excluded from this analysis.

#### Study design

This was a cross-sectional study with 14 schools selected from a list of 107 schools obtained from the Western Cape Education Department. Learners were recruited through a proportionally stratified multistage random sampling technique which occurred at the class and learner level [14]. Learners with a history of diabetes or without consent were excluded.

After explaining all the procedures, written informed consent from parents and oral assent from students was obtained [14]. Permission to conduct the study was also obtained from the Western Cape Department of Education, school governing bodies and school principals. The study obtained ethical approval from the Faculty of Health and Wellness Sciences Ethics Committee, Cape Peninsula University of Technology and was conducted according to the Code of Ethics of the World Medical Association (Declaration of Helsinki).

### Data collection

#### Interviews

Qualified healthcare professionals conducted all interviews in the language of the learner i.e. English, Afrikaans and Xhosa. Data were collected on physical activity and eating habits. Physical activity and the dietary behaviours were assessed using an adaptation of the validated questionnaire designed by Arvidsson and colleagues [15]. The frequency per week at which learners performed activities such as walking and sport defined physical activity levels while for sedentary behaviour, time spent watching television was used as a proxy. Physical inactivity was defined as ≤2 days of activity per week and sedentary behaviour as 3–5 days of television watching per week.

Food groups that were consumed the previous day as well as whether the learner had eaten breakfast were recorded. The nine food groups that were assessed were: cereals, roots & tubers; meat, poultry & fish; dairy; eggs; vitamin A-rich fruit & vegetables; legumes, nuts& seeds; other fruits; other vegetables; oils & fats. A dietary diversity score (DDS) was calculated by allocating a point to each food group. A score below 4 defined poor dietary diversity. Furthermore, learners were asked if they generally smoked and consumed alcohol.

### Clinical measurements

Blood pressure was measured using a semi-automatic digital blood pressure monitor (Rossmax PA, USA), following the WHO guidelines [14], on the right arm in a sitting position after a 10 min rest period. Three readings were taken at five minute intervals and the lowest of the three readings was used in the current analyses. Hypertension was defined as systolic and/or diastolic blood pressure equal or greater than the 95th percentile for gender, age and height [16].

Weight, to the nearest 0.1 kg was determined with the subject in light clothing and without shoes and socks, using a Sunbeam EB710 digital bathroom scale, which was calibrated and standardized using a weight of known mass. Height, to the nearest 0.1 cm was recorded using a stadiometer with participants standing on a flat surface at a right angle to the vertical board of the stadiometer. All anthropometric measurements were performed three times and the average used for analysis. Body mass index (BMI) was calculated as weight per square metre of height (kg/m^2^). Overweight and obesity status were assessed using age-gender-specific cut-off points from international references provided by the International Task Force as developed by Cole and colleagues [17].

### Biochemical analyses

Finger prick blood was used for the estimation of lipid levels using the CardioCheckTM P.A analyzer (Polymer Technology Systems, Inc. USA). The commercial glucometer used in this study had a mean imprecision of <5%, with a range of 1.1–33.3 mmol/L on capillary whole blood [14]. Dyslipidaemia was defined as follows: total cholesterol >5 mmol/L, triglycerides >1.5 mmol/L, high-density lipoprotein cholesterol (HDL-C) <1.2 mmol/L [18].

### Statistical analysis

Analyses were done using the statistical package STATA 13. Socio-demographic characteristics were summarised as count and percentages for categorical variables and means and standard deviations (SD) for continuous variables. Group comparisons for categorical variables used chi square tests and for continuous variables, the Student’s t-test and analysis of the variance (ANOVA) were used. Multivariable logistic regressions were performed to assess the associations between the socio-demographic and behavioural factors, and overweight/obesity with adjustments for age, ethnicity and gender. Multivariable logistic regressions were also performed to assess the associations between the cardio-metabolic comorbidities and overweight/obesity with adjustments for age, ethnicity and gender. The number of statistical tests performed mean that it is highly likely that at least some significant findings may be due to random associations. All the statistical tests were performed at a 5% level of significance.

## Results

### Socio-demographic and behavioural characteristics

Of the 1960 learners invited to participate, 1559 were included in this study with a response rate of 79.5%. Table 1 presents the baseline characteristics stratified by gender and population group. The majority of participants were female (60.3%) and consisted of 145 White, 537 Black, and 877 Coloured learners. The mean age of 12.9 years (SD = 2.5) was similar in boys and girls (*p* = 0.226) but significantly different by population group with White learners being the oldest (*p* < 0.0001). All White learners but only about half (53.2%) of Coloured and 61.5% of Black learners lived in urban areas.

**Table 1:** Socio-demographic and behavioural characteristics of participants presented by gender, population group and overall

Characteristics	Overall	Gender			Population groups				*P* for population group* gender*
		Male	Female	*P*-value	Coloured	Black	White	*P*-value	
Number, *n* (%)	1559 (100)	619 (39.7)	940 (60.3)		877 (56.3)	537 (34.5)	145 (9.3)		
Mean age, years (SD)	12.9 ± 2.5	13.0 ± 2.6	12.8 ± 2.5	0.226	12.7 ± 2.6	12.9 ± 2.5	14.0 ± 2.0	<0.0001	0.001
Age group (years), *n* (%)				0.165				<0.0001	<0.0001
7–9	185 (11.9)	77 (12.4)	108 (11.5)		127 (14.5)	51 (9.5)	7 (4.8)		
10–12	483 (31.0)	172 (27.8)	311 (33.1)		285 (32.5)	183 (34.1)	15 (10.3)		
13–15	626 (40.1)	257 (41.5)	369 (39.2)		317 (36.1)	209 (38.9)	100 (69.0)		
16–18	265 (17.0)	113 (18.3)	152 (16.2)		148 (16.9)	94 (17.5)	23 (15.9)		
Location, *n* (%)				0.043				<0.0001	<0.0001
Rural	142 (9.5)	44 (7.4)	98 (10.9)		138 (16.7)	4 (0.7)	-		
Semi-urban	448 (29.9)	197 (33.1)	251 (27.8)		249 (30.1)	199 (37.8)	-		
Urban	908 (60.6)	355 (59.5)	553 (61.3)		441 (53.2)	324 (61.5)	143 (100)		
Behavioural risk factors*,* *n* (%)									
Physical inactivity**,	423 (27.1)	159 (25.7)	264 (28.1)	0.297	252 (28.7)	119 (22.2)	52 (35.9)	0.001	0.003
Sedentary behaviour***,	1303 (83.6)	507 (81.9)	796 (84.7)	0.004	709 (80.8)	469 (87.3)	125 (86.2)	0.148	0.007
Smoking	122 (7.9)	71 (11.5)	51 (5.4)	<0.0001	85 (9.9)	16 (3.0)	21 (14.5)	<0.0001	<0.0001
Alcohol consumption	223 (14.4)	111 (17.9)	112 (11.9)	0.001	128 (14.7)	51 (9.6)	44 (30.3)	<0.0001	<0.0001
Dietary Diversity Score ≤ 4	667 (43.8)	269 (44.6)	398 (43.3)	0.616	371 (43.7)	273 (51.6)	23 (16.0)	<0.0001	<0.0001

21 missing (10 males; 11 females); (15 Coloured; 6 Black learners)

*P-value for the interaction of population group and gender; **participating in sport and/or walking on ≤2 days/week; ***television watching 3–5 days/week

Physical inactivity levels were significantly higher in Whites (35.9%) than in Coloured (28.7%) and Black learners (22.2%) but similar by gender. Sedentary behaviour was extremely high at 83.6% with females more sedentary than males (84.7% vs. 81.9%, *p* = 0.004). Of concern was that 7.9% and 14.4% of learners smoked and drank alcohol with the highest prevalence in White learners at 14.5% and 30.3%, respectively. More boys than girls consumed alcohol (17.9% vs. 11.9%, *p* = 0.001) and smoked (11.5% vs. 5.4%, *p* < 0.0001).

Regarding dietary diversity, 43.8% of learners consumed ≤4 food groups per day with poor DDS most prevalent in Black (51.6%) and Coloured (43.7%) compared with White learners (16%). There were significant gender*ethnicity interactions in the distribution of all baseline characteristics (Table 1).

### Prevalence of overweight and obesity

The overall overweight and obesity prevalence was 15.6% and 7.3%, respectively, with significantly higher levels in girls (19.7% and 9.1%) than in boys (9.4% and 4.5%), *p* < 0.0001(Table 2). By population group, White learners were the most overweight (20.7%) while obesity was most prevalent in Coloured learners (7.5%). There was a significant gender*ethnicity interaction in the distribution of BMI categories (*p* < 0.0001).

**Table 2:** Prevalence of adiposity and cardio-metabolic comorbidities by gender, population group and overall

Variables, *n* (%)	Overall	Gender			Population groups				P for population group* gender*
		Boys	Girls	*P*-value	Coloured	Black	White	*P*-value	
*N*, %	1559 (100)	619 (39.7)	940 (60.3)		877 (56.3)	537 (34.5)	145 (9.3)		
Body Mass Index categories				<0.0001				<0.0001	<0.0001
Normal weight	1202 (77.1)	533 (86.1)	669 (71.2)		699 (79.7)	398 (74.1)	105 (72.4)		
Overweight	243 (15.6)	58 (9.4)	185 (19.7)		112 (12.8)	101 (18.8)	30 (20.7)		
Obesity	114 (7.3)	28 (4.5)	86 (9.1)		66 (7.5)	38 (7.1)	10 (6.9)		
Hypertension	38 (2.6)	17 (2.9)	21 (2.4)	0.748	17 (2.0)	18 (3.6)	3 (2.2)	0.038	0.072
Dyslipidaemia									
Triglycerides >1.5 mmol/L	78 (5.0)	26 (4.2)	52 (5.5)	0.238	42 (4.8)	29 (5.4)	7 (4.8)	0.873	0.040
Total cholesterol >5 mmol/L	102 (6.5)	28 (4.5)	74 (7.9)	0.009	61 (7.0)	27 (5.0)	14 (9.7)	0.102	0.034
HDL-C < 1.2 mmol/L	1047 (67.2)	459 (74.2)	588 (62.6)	<0.0001	593 (67.6)	339 (63.1)	115 (79.3)	0.001	<0.0001

*P-value for the interaction of population group and gender; Overweight and obesity: Gender- and age-specific BMI cut-off points corresponding to 25 and 30 kg/m2, respectively, at the age of 18 years (17); Hypertension: systolic and/or diastolic blood pressure equal to the 95th percentile for gender, age and height (16)

### Prevalence of cardio-metabolic comorbidities

For the cardio-metabolic risk factors, the prevalence was as follows: hypertension: 2.6%, hypercholesterolaemia: 6.5%, hypertriglyceridaemia: 5% and low HDL-C: 67.2%. By gender, hypertension (2.9% vs. 2.4%) and hypertriglyceridaemia (4.2% vs. 5.5%) were similarly distributed between boys and girls (all *p* > 0.238), Table 2. Low HDL-C (74.2% vs. 62.6%) but not hypercholesterolaemia (4.5% vs. 7.9%) was more frequent in boys than in girls (all *p* < 0.009). Across population groups, there were significant differences for hypertension which was most frequent in Black learners (*p* = 0.038) and for low HDL-C levels which was most prevalent in White learners (*p* = 0.001).

### Socio-demographic and behavioural associations with overweight/obesity

The associations of the socio-demographic and behavioural characteristics with overweight/obesity are presented in Table 3. In the univariable analysis, gender (*p* < 0.0001), population group (*p* = 0.019) and physical activity (*p* = 0.040) were associated with overweight/obesity. In age, gender and ethnicity adjusted models, being female (2.53; 1.94–3.32), Black (1.33; 1.02–1.72) or physically inactive (1.32; 1.01–1.72) significantly increased the likelihood of being overweight/obese. Expansion of the model to include the other covariates did not affect the associations.

**Table 3:** Stepwise multivariable logistic regression model for the associations of socio-demographic and behavioural factors with overweight/obesity

Variables	Basic model			Expanded model		
	OR	95% CI	*P*-value	OR	95% CI	*P*-value
Gender						
Boys	1			1		
Girls	2.53	1.94–3.32	<0.0001	2.51	1.92–3.29	<0.0001
Age groups, years			0.325			0.325
7–9	1			1		
10–12	1.16	0.75–1.80	0.509	1.21	0.78–1.87	0.404
13–15	1.36	0.89–2.07	0.161	1.39	0.91–2.12	0.132
16–18	1.40	0.87–2.24	0.167	1.45	0.90–2.33	0.130
Population groups			0.019			0.019
Coloured	1			1		
Black	1.33	1.02–1.72	0.032	1.35	1.04–1.75	0.024
White	1.48	0.98–2.25	0.063	1.45	0.96–2.21	0.080
Region			0.540			
Rural	1					
Semi-urban	1.36	0.81–2.25	0.242	-	-	-
Urban	1.50	0.89–2.53	0.130	-	-	-
Eating breakfast						
No	1					
Yes	0.83	0.63–1.08	0.165	-	-	-
Physical inactivity						
No	1			1		
Yes	1.32	1.01–1.72	0.047	1.32	1.01–1.72	0.047
Sedentary behaviour						
No	1					
Yes	1.21	0.86–1.70	0.284	-	-	-
Smoking						
No	1					
Yes	0.80	0.48–1.33	0.396	-	-	-
Alcohol						
No	1					
Yes	0.97	0.67–1.41	0.873	-	-	-

Gender: adjusted for age and population groups; Age group: adjusted for gender and population group; Population group: adjusted for gender and age group; Physical inactivity: adjusted for gender, age and population groups; Physical inactivity: Sport and/or walking ≤2 days/week

### Cardio-metabolic associations with overweight/obesity

Table 4 shows the associations of the other cardio-metabolic diseases with overweight/obesity. In the overall model, adjusted for age, gender and population group, hypertension (3.02; 1.52–6.0), hypertriglyceridemia (1.83; 1.12–2.99), and low HDL-C (2.53; 1.89–3.40) were associated with increased odds of being overweight/obese. In the regression analyses stratified by population group and adjusted for age and gender, the odds for hypertension were associated with overweight/obesity in coloured (3.27; 1.18–9.08) and black (3.62; 1.31–10.04) learners. The likelihood for low HDL-C with overweight/obesity was five-fold higher in White learners, almost four-fold higher in Coloured learners and 1.5 times greater in Black learners.

**Table 4:** Odds ratios (OR) and 95% confidence intervals (CI) for the associations of cardio-metabolic comorbidities with overweight/obesity

Outcomes	Overall^a^		Population groups^b^					
			Coloured		Black		White	
	OR (95% CI)	*P*-value	OR (95% CI)	*P*-value	OR (95% CI)	*P*-value	OR (95% CI)	*P*-value
Hypertension								
No	1		1		1		1	
Yes	3.02 (1.52–6.00)	0.002	3.27 (1.18–9.08)	0.023	3.62 (1.31–10.04)	0.013	1.63 (0.14–19.52)	0.702
Triglycerides > 1.5 mmol/L								
No	1		1		1		1	
Yes	1.83 (1.12–2.99)	0.016	1.94 (0.99–3.78)	0.053	1.22 (0.52–2.88)	0.642	4.0 (0.79–20.21)	0.095
Total cholesterol > 5 mmol/L								
No	1		1		1		1	
Yes	1.04 (0.65–1.68)	0.869	0.61 (0.29–1.28)	0.189	1.86 (0.82–4.19)	0.137	1.40 (0.43–4.56)	0.574
HDL-C < 1.2 mmol/L								
No	1		1		1		1	
Yes	2.53 (1.89–3.40)	<0.0001	3.65 (2.33–5.72)	<0.0001	1.56 (1.01–2.40)	0.046	5.04 (1.35–18.80)	0.016

^a^Adjusted for age, gender and population group; ^b^Adjusted for age and gender; Hypertension: systolic and/or diastolic blood pressure equal to the 95th percentile for gender, age and height

In the regression analyses, adjusted for age, gender and population group, systolic and diastolic blood pressure, low HDL-cholesterol and high triglycerides were statistically significant associated with an increased risk of overweight/obesity (Additional file 1: Table S1).

## Discussion

This study assessed the prevalence of overweight and obesity, and its associations with socio-demographic characteristics, behavioural risk factors and other cardio-metabolic diseases among school learners in the Western Cape, South Africa. It highlights the high burden of childhood and adolescent overweight/obesity in South Africa which is a reflection of the trend occurring worldwide, including in low and middle-income countries [19, 20]. Over one in five learners in this study were overweight/obese with girls more likely to be overweight/obese than boys. The overweight/obesity prevalence in girls (28.8%) is comparable with the 26.3% prevalence reported in the Global Burden of Disease estimates for girls <20 years of age in South Africa in 2013 [19]. However, the prevalence in boys was slightly lower in this study (13.9%) compared with the national estimates (18.8%) [19].

The higher prevalence of overweight/obesity in girls compared with boys has been reported in numerous South African studies [5, 21], as well as in studies in Sub-Saharan Africa [22]. Rossouw et al. [5] postulated that these gender differences may be due to potential differences in the energy needs between boys and girls, in the timing of sexual maturation, and in the levels of physical activity. The gender difference in overweight/obesity prevalence reflects the adult patterns in the country where women have markedly higher levels than men. However, the significant gender*ethnicity interactions for overweight/obesity found in this, as well as in previous studies [23, 24], demonstrate associations between these factors and overweight/obesity.

As in other studies [23, 25], differences in overweight/obesity prevalence by population group were evident in our study, whereby black and white South African children had higher obesity rates than the coloured group. Numerous factors are known to contribute to these population differences including genetics and environmental influences [26] such as different dietary habits [27], and varied cultural, socioeconomic and family factors [28]. Notably, perceptions of body image ideals differ markedly with bigger body sizes commonly linked to health, wealth and happiness, preferred by Black South African females [29].

The association of physical inactivity with overweight/obesity accords with previous studies [25, 30, 31]. However, unlike other reports, sedentary behaviour was not significantly related to overweight/obesity in this study. This may be due to the measure used in this study, television viewing frequency which may not be an appropriately sensitive proxy in the local setting.

Interestingly, previous South African studies have reported that urban residence is associated with an increased risk of overweight/obesity [5, 25, 32]. This may be an indication of the uptake of urban lifestyle behaviours relating to diet and physical activity patterns in rural areas. However, our study has been unable to demonstrate significant relationships between this risk factor and overweight/obesity which can be explained by the small sample size used in this study and the consequent low statistical power to detect these associations.

Despite their youth, the cardio-metabolic diseases of hypertension and dyslipidaemia that are commonly linked to overweight/obesity were already prevalent in these schoolchildren. This highlights that these cardio-metabolic comorbidities do not only occur with older age but may develop in the young, particularly in the presence of overweight/obesity. These findings are consistent with other studies, which found a strong link between the development of overweight and obesity and cardio-metabolic comorbidities [33–35]. Therefore, if the current high burden of childhood overweight/obesity is not addressed, it is likely that cardio-metabolic comorbidities, once a preserve of the elderly, may become more prevalent in the young. This may lead to cardiovascular events occurring earlier, possibly even in the third and fourth decades of life, with devastating consequences including a potential decrease in longevity.

The differential associations of cardio-metabolic comorbidities with overweight/obesity across population groups demonstrated in this study are in line with other reports [34, 36, 37]. Whincup et al. [37] reported differences in CVD risk factors and vascular disease markers in British children of South Asian, African-Caribbean and European origin. It can thus be suggested that cardiovascular disease prevention needs to take into account these ethnic disparities by not only addressing the disease burden, but also the socioeconomic and cultural background of the different ethnic groups.

### Strengths and limitations of the study

The key strength of the present study is the representativeness of the sample with the inclusion of learners from three South African population groups who resided in both urban and rural areas. These findings may be generalised to other South African schoolchildren, particularly in lower- and middle- income communities. However, learners from higher-income households attending private schools were not included in this study and these findings are unlikely to be generalizable to them. Among the study limitations is the cross-sectional design which precludes inferences of causal associations. The use of self-reported rather than objectively measured ambulation or physical activity and sedentary behaviour is likely to have been subjected to recall bias and measurement error. Sedentary behaviour in our study was based on time watching TV per week which may provide less accurate results than the average time of the TV watching. The self-reported histories of tobacco and alcohol use may have resulted in an underestimation of these risky behaviours in learners. The classification in different population group was self-reported and may have introduced bias [38]. The socio-cultural and psychological influences of overweight/obesity, as well as the learners‘socioeconomic positions, which play a pivotal role in the local context, were not examined in this study and is another limitation. At last, the main limitation is that the number of statistical tests that were conducted in our study mean that it is highly likely, that at least some significant findings may be due to random associations.

## Conclusions

This study highlights the greater likelihood for overweight/obesity in female and Black learners in the Western Cape. Therefore, it is important to develop overweight/obesity prevention and intervention programmes that target these specific sub-groups. Intervention strategies need to take cognisance of gender differences and cultural perceptions, particularly the positive connotations linked to overweight/obesity in Black females. The association of cardio-metabolic comorbidities with overweight/obesity in children and adolescents emphasises the urgency of early interventions to curb the uptake of this condition in childhood. Moreover, it underscores the need for a comprehensive clinical assessment in the overweight/obese child. Further research is required for an in-depth understanding of the role, if any, played by physical activity in the development of overweight/obesity as well as on the patterns of sedentary behaviours and in this community. Such information will enable the development of appropriate obesity prevention and intervention programmes.

## Additional file

Additional file 1: Table S1. Multivariable logistic regression for cardio-metabolic associations with overweight/obesity (DOCX 23 kb)

## Abbreviations

ANOVA: Analysis of the variance
BMI: Body mass index
BPs: Blood pressures
CVDs: Cardiovascular diseases
DDS: Dietary diversity score
HDL-C: High-density lipoprotein cholesterol
LDL-C: Low-density lipoprotein cholesterol
SD: Standard deviations
