# Supplementary material for: Differential prevalence and associations of overweight and obesity by gender and population group among school learners in South Africa: a cross-sectional study
Source: BMC Obes. 2017 Jul 17;4:29. doi: 10.1186/s40608-017-0165-1 (PMC5514529; doi:10.1186/s40608-017-0165-1)
Supplement: Additional file 1: Table S1. — Multivariable logistic regression for cardio-metabolic associations with overweight/obesity (DOCX 23 kb) [file 40608_2017_165_MOESM1_ESM.docx]

Supplementary Material

Differential prevalence and associations of Overweight and Obesity by gender and population group among School Learners in South Africa: A cross-sectional study

S. Negash, C Agyemang, T.E. Matsha, N Peer, R.T. Erasmus, A.P. Kengne*

*** Correspondence:** Corresponding Author: andre.kengne@mrc.ac.za

# Supplementary Tables

**Supplementary Table 1: Multivariable logistic regression for cardio-metabolic associations with overweight/obesity**

| **Variables** | **Basic model*** | | |
| --- | --- | --- | --- |
|  | **OR** | **95%CI** | **P-value** |
| **Systolic BP** | 1.05 | 1.04-1.06 | <0.0001 |
| **Diastolic BP** | 1.05 | 1.04- 1.07 | <0.0001 |
| **High total cholesterol** | 1.10 | 0.94- 1.26 | 0.245 |
| **Low HDL-cholesterol** | 0.28 | 0.19- 0.41 | <0.0001 |
| **High triglycerides** | 1.50 | 1.20-1.87 | <0.0001 |

*Adjusted for age, gender and population group
